# Tinea Sycosis; with Report of Case*Read at meeting of West Texas Medical Society, July 31, 1902.

**Published:** 1902-10

**Authors:** Alexander A. Brown

**Affiliations:** San Antonio, Texas, Late Resident Physician of the Jefferson Medical College Hospital of Philadelphia


For Texas Medical Journal.

### Tinea Sycosis; With Report of Case.*

BY ALEXANDER A. BROWN, M. D., SAN ANTONIO, TEXAS,
Late Resident Physician of the Jefferson Medical College Hospital of Philadelphia.

Tinea sycosis is a parasitic skin affection belonging to the worm group. The specific organism is the trichophyton fungus. Its sites of predilection are the chin, neck and bearded portion of the cheek. The upper lip is rarely, if ever, involved in this affection, this making an important point in differential diagnosis between this and non-parasitic sycosis. The infection may remain superficial, resembling the tinea of the scalp in appearance, but ordinarily develops into its classical type. The organism attacks the hair and hair follicles of the before mentioned regions. When the disease is first seen it may have the appearance of small, reddish, scaly patches, but the infection soon causes induration which becomes distinctly nodular and lumpy; the involved hairs become dry and brittle. break off, and frequently fall out. If not, they are so loosely held as to be easily removed without pain or discomfort to the patient. Pustules occur at the root of the hair follicles which usually break, emitting a yellow pus which quickly dries and forms crusts on the surface. The subjective symptoms are but slight, the patient complaining of some itching and burning. These symptoms, however, may be much intensified.

The source of infection is usually from barber shops, but may be contracted from other sources, as was the case which I had under observation, which I believe had its origin from one or more of the cattle which the patient had under his care.

The disease with which parasitic sycosis is most likely to be confounded is simple sycosis. The differential diagnosis, however, is usually easy. The upper lip is attacked by simple sycosis, but not by the parasitic. The lumpiness and nodular tumefactions are not found in simple sycosis, while the pustules in the simple form are small and discrete, and in the parasitic they are larger, breaking

---

*Read at meeting of West Texas Medical Society, July 31, 1902.

and forming crusts. In the parasitic variety the hairs are brittle, breaking off, frequently falling out, and are but loosely held, while in simple form, the hairs are firmly held until free suppuration takes place.

Parasitic sycosis is rapid in progress, marked changes being visible in a short time; while in the other form the course is slow, its appearance changing but little from week to week. By microscopical examination we find in one the trichophyton fungus and its spores in the hairs; in the other it is absent.

The course of this disease is chronic and progressive. By diligent treatment cure may be effected in from one to two or three months. Relapses are likely to occur.

The history of the case is as follows:

C. C., age 22, male, married, farmer by occupation. Has had the ordinary diseases of childhood. At present enjoys good general health. States that no one in his family has an eruption on face; also does not remember having come in contact with any one suffering with a similar condition or using infected linen. Has never been shaved in a barber shop. Patient has the care of a large number of horses and cattle with which he comes in daily contact.

Two months before consulting me, he noticed several rounded, scaly patches of a reddish color on his chin. These gradually became more numerous and increased in size, the sites of the eruption becoming lumpy and nodular and numerous pustules forming on the infected areas.

When I saw patient the disease was of two months' standing. Its appearance was a typical picture of tinea sycosis. The affected areas were lumpy and nodular, with crust formation and numerous pustules. The hairs were brittle and a number broken off. The patient complained of itching and burning which was not severe.

*Treatment.*—The crusts were softened with a bland oil and then the parts washed with warm water and tar soap. The involved hairs were removed each day with thumb forceps. The parts were thoroughly cleansed each morning and night with tar soap and warm water and hot cloths applied for ten minutes. A solution consisting of tinc. myrrh one ounce, hydrarg. chlor. corr. one gr. was thoroughly painted over the affected area immediately after application of hot cloths in the morning. At night an ointment consisting of hydrarg. oleat. two drachms, acid carbol. thirty grs., petrolati two ounces was applied.

One week after treatment was instituted the improvement was sufficiently marked to be noticed by the patient, and at the end of four weeks all evidence of the disease had disappeared.

